# Cases of Delirium Tremens, Successfully Treated by the Administration of Chloroform

**Published:** 1852-05

**Authors:** Stephen H. Pratt

**Affiliations:** Baltimore


From the Amer. Jour. of Med. Science.

*Cases of Delirium Tremens successfully treated by the administration of Chloroform.* By STEPHEN H. PRATT, M. D., of Baltimore.

CASE I. May 7th, 1850, I called to see E. B., laboring under delirium tremens.

E. B. had, that day, been taken from the ——— Infirmary, where he had been for the last seven days under judicious treatment for the above-named disease. During the time (seven days) he had not slept any, as I had been, that morning, informed by the resident physician; and his case was deemed almost hopeless. His friends became alarmed, and (very injudiciously, I thought) removed him, and placed him under my care.

It was 1 o'clock, P.M., when I saw him. He was very feeble and much exhausted by disease and protracted wakefulness. His pulse was feeble and frequent. There was subsultus, muttering, great incoherence, with cold and clammy extremities.

Having been advised that he had been on a mixed opiate and stimulant treatment, at least a part of the time, and having had some success previously in the use of chloroform, I determined to use it now. Accordingly one drachm of chloroform, diluted with water, was exhibited. At 5 o'clock, P.M., another drachm was administered; and at 9 still another, diluted as before. At 10, he fell asleep and slept till morning. At 8, in the morning, he waked and drank some gruel, after which he soon fell asleep and slept till noon.

He now waked with a good appetite, which he too freely indulged by partaking of soup. However, he was quite comfortable during the afternoon, and slept well through the night. Next morning he vomited two or three times freely. The emesis was not violent, and was easily controlled. From this time, paying strict attention to his diet, he rapidly convalesced.

During his sickness no medicine was exhibited but chloroform (not even aperients), and this but three times. On the fifth day, the patient left the house to attend to his affairs, and was soon in health.

CASE II. Was called to see J. H., June 4th, 1851, laboring under delirium tremens. Put him upon a mixed opiate and stimulant treatment through the day, and exhibited opium in full doses through the night. This was continued two days and nights, without benefit. Indeed the patient grew worse. The third morning I put him upon: ℞. Spts. sulph. ætheris comp. tinct. valerianæ, ana ℥iss.; to take ʒii. every two or three hours, intermediately giving tinct. opii. At 8 P.M., gave a large opiate. At 10 P.M., gave tinct. opii. ʒj. At 12, repeated the dose; and at 2, again repeated it. All this time the patient grew worse, and became "furiously delirious," frightening all the household.

Three men were appointed to prevent him from jumping out of the windows (several attempts at which he had made), or otherwise injuring himself. At times he was a match for them all. At length he grew weak, becoming more and more prostrated by his great exertions. The family became alarmed, and wished further advice. A consulting physician was called in. A hot stimulating pediluvium and an opio tartar emetic treatment was agreed upon.

I suggested chloroform internally, which was not wholly objected to, though not preferred by the consulting physician. Accordingly the former was tried, but unfortunately without success, the patient rapidly growing worse.

He was now beyond control, a raving maniac, a terror to all present. His pulse was feeble and frequent; so frequent it could not be counted with the existing tremor. His tongue was dry; there was also muttering, subsultus, and perfect incoherence, with cold and clammy extremities.

Under these circumstances, I determined to exhibit chloroform as a dernier resort. A tea-spoonful nearly, diluted with water, was administered. After one hour, the following was given: ℞. Spts. sulph. ætheris comp. tinct. valeriana, aa f ʒii., chloroform f ʒi., at a draught.

(The compound spirit of sulphuric ether and tinct. valerian were added in order to obviate, if possible, the danger of fatal prostration.) Fifteen minutes after its exhibition, the patient fell asleep, and slept soundly three and a half hours. Meantime, perspiration ceased; his extremities became *warm;* and his pulse grew *calmer, fuller* and *firmer*. He then awoke much refreshed and quite rational, and had a free, natural dejection.

Three tea-spoonsful of the mixture, ℞. Hoff.'s anodyn. and tinct. valeriana, with half a tea-spoonful of chloroform, were then exhibited. After this, he washed his hands and face, and bathed himself generally. In one hour, I exhibited f ʒ iv. of the mixture, with f ʒ i. of chloroform, and persuaded him to lie down. In a few minutes he was asleep, and slept comparatively soundly four hours, when he arose, went down stairs, and evacuated his bowels. In fifteen minutes he was again asleep, and slept three hours, when he waked and drank a tumbler of milk, took a dose of spts. sulph. ætheris comp. and tinct. valeriana; fifteen minutes afterwards he was asleep again, and continued sleeping through the night, rising, meantime, but once.

In the morning he rose, drank some milk and beef tea, and after evacuating his bowels again went to sleep. His pulse was now good; extremities warm, glowing; subsultus greatly diminished; delirium almost entirely wanting. He slept till about noon, and then waked still more tranquil. During the afternoon, he slept and waked alternately, and rested well the following night. His sleep was not comatose. When awake, he was wide awake, cheerful and lively. A day or two passed thus as he rapidly convalesced. On the 9th, he was walking about the city a comparatively well man. He has continued well since.

Such are the facts. From a furious delirium, with subsultus, perfect incoherence, cold, clammy extremities, a feeble, fluttering, frequent pulse, costiveness, &c., by the tranquilizing and peculiar (shall I say specific?) influence of chloroform, he was *rescued*, in a little more than an hour, and thrown into a condition the most favorable possible; from which in a few days, he was restored to his usual health. No emesis, or irritation of the bowels, occurred. No cathartics were exhibited, yet gentle motions followed the administration of chloroform.

The methodus medendi of this wonderful agent, I will not here attempt to explain. Facts are of more importance than inferences, and if, by this contribution, I add one to the *facts* already recorded, I shall be satisfied.

